# A Novel Constitutional t(3;8)(p26;q21) and *ANKRD26* and *SRP72* Variants in a Child with Myelodysplastic Neoplasm: Clinical Implications

**DOI:** 10.3390/jcm12093171

**Published:** 2023-04-28

**Authors:** Viviane Lamim Lovatel, Ana Paula Bueno, Elaiza Almeida Antônio de Kós, Laura Guimarães Corrêa Meyer, Gerson Moura Ferreira, Mayara de Fátima Kalonji, Fabiana Vieira de Mello, Cristiane Bedran Milito, Elaine Sobral da Costa, Eliana Abdelhay, Maria Dolores Tabernero Redondo, Maria S. Pombo-de-Oliveira, Teresa de Souza Fernandez

**Affiliations:** 1 Cytogenetic Laboratory, Cell and Gene Therapy Program, Instituto Nacional do Câncer (INCA), Rio de Janeiro 20230-130, Brazil; 2 Instituto de Puericultura e Pediatria Martagão Gesteira, Universidade Federal do Rio de Janeiro, Rio de Janeiro 21941-909, Brazil; 3 Pathology Department, Federal University of Rio de Janeiro, Rio de Janeiro 21941-599, Brazil; 4 Outpatient Department, Bone Marrow Transplantation Center, Instituto Nacional de Câncer, Rio de Janeiro 20230-130, Brazil; 5 Stem Cell Laboratory, Bone Marrow Transplantation Center, Instituto Nacional de Câncer, Rio de Janeiro 20230-130, Brazil; 6 Instituto de Investigación Biomédica de Salamanca (IBSAL), 37007 Salamanca, Spain; 7 Research Centre, Instituto Nacional de Câncer (INCA), Rio de Janeiro 20231-050, Brazil

**Keywords:** childhood myelodysplastic neoplasm, constitutional cytogenetic abnormality, genetic variants

## Abstract

Background: Childhood myelodysplastic neoplasm (cMDS) often raises concerns about an underlying germline predisposition, and its verification is necessary to guide therapeutic choice and allow family counseling. Here, we report a novel constitutional t(3;8)(p26;q21) in a child with MDS, inherited from the father, the *ANKRD26* and *SRP72* variants from the maternal origin, and the acquisition of molecular alterations during MDS evolution. Case presentation: A 4-year-old girl showed repeated infections and severe neutropenia. Bone marrow presented hypocellularity with dysplastic features. The patient had a t(3;8)(p26;q21)c identified by G-banding and FISH analysis. The family nucleus investigation identified the paternal origin of the chromosomal translocation. The NGS study identified *ANKRD26* and *SRP72* variants of maternal origin. CGH-array analysis detected alterations in *PRSS3P2* and *KANSL* genes. Immunohistochemistry showed abnormal p53 expression during the MDS evolution. Conclusion: This study shows for the first time, cytogenetic and genomic abnormalities inherited from the father and mother, respectively, and their clinical implications. It also shows the importance of investigating patients with constitutional cytogenetic alterations and/or germline variants to provide information to their family nucleus for genetic counseling and understanding of the pathogenesis of childhood MDS.

## 1. Introduction

According to the 5th edition of the World Health Organization (WHO) classification, the myelodysplastic syndrome is now replaced by myelodysplastic neoplasm (MDS) [1]. Childhood myelodysplastic neoplasm (cMDS) is a rare disease, accounting for less than 5% of childhood hematologic malignancies, and it is associated with an elevated risk of evolution to acute myeloid leukemia (AML) [2,3].

The cMDS often raises concerns about germline predisposition, observed in 15% of MDS diagnoses [3]. Some family members with germline variants present a silent clinical picture and do not develop hematologic malignancy, while others develop after decades due to the acquisition of new alterations [3,4]. The penetrance of these germline alterations is heterogeneous. The progression to myeloid neoplasm is generally due to the acquisition of an additional mutation, somatic changes, and/or loss of heterozygosis [3,4,5,6]. Thus, the verification of germline alterations is necessary to inform therapeutic considerations, guide pre- and post-treatment follow-up, and allow for family counseling.

Among the genes already described in MDS with germline origin, the following can be highlighted: transcriptional regulators such as *CEBPA*, *RUNX1*, *ETV6*, and *GATA2*; telomerase regulators *TERC*/*TERT* and splicing and signal transduction controllers *SAMD9*/*SAMD9L*, *RAS*/*MAPK*, and *DDX41*; kinase signal regulators like *ANKRD26*; and protein translocation and processing regulators like *SRP72* [3,4,5,6,7].

Constitutional chromosomal abnormalities have an increased risk of developing MDS/AML, leading to genetic instability that may reflect changes in cell proliferation and differentiation [3,8]. Some constitutional alterations such as trisomy 8, inversion of 3, duplication of 1q, Robertsonian translocations, and/or trisomy 21 (Down Syndrome) have been described in patients who develop AML/MDS [9,10,11,12]. Nevertheless, it has been demonstrated that the presence of constitutional abnormalities is not sufficient for full neoplastic transformation, and it is necessary for the acquisition of other molecular abnormalities [13]. However, how many and which cytogenetic and molecular abnormalities are required for this neoplastic process remain unresolved questions. Here, we describe a yet unreported t(3;8)(p26;q21) constitutional difference associated with molecular hits, *ANKRD26* and *SRP72* germline variants, cryptic chromosomal alterations in *PRSS3P2* and *KANSL1* genes, and abnormal p53 expression, leading to cMDS development and clinical progression.

## 2. Case Presentation

### Clinical and Laboratory Analysis

**Figure 1:**
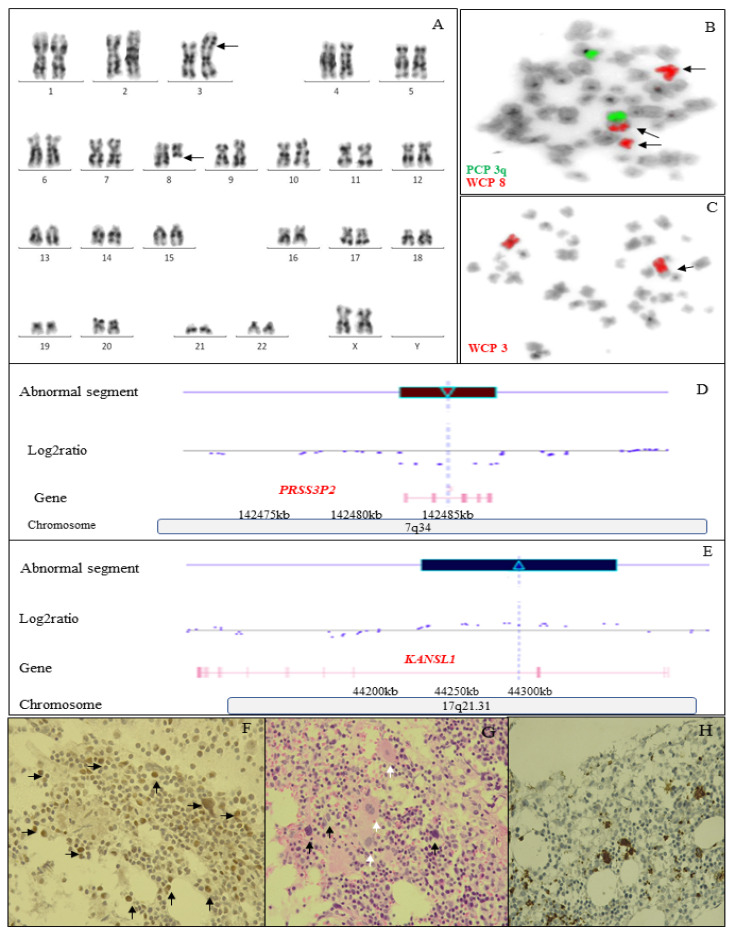
Cytogenetic and molecular alterations in a pediatric patient with MDS. (**A**) Cytogenetic analysis by G-banding showed the karyotype 46,XX,t(3;8)(p26;q21)c; the arrows indicate the chro-mosomal location where the translocation occurred. (**B**) FISH analysis using partial chromosome painting probe (PCP) for chromosome 3q (green color) and WCP for chromosome 8 (red color) showed that only one chromosome 8 segment were translocated to chromosome 3, showing that it is not a reciprocal translocation; the arrows indicate chromosome 8 at three times, normal chromo-some 8, the deleted chromosome 8, and the chromosome 8 segment translocated into chromosome 3. (**C**) FISH analysis using WCP for chromosome 3 (red color) confirming that chromosome 3 is not translocated; the arrow indicate chromosome 3 where the translocation of chromosome 8 occurred. (**D**) Array-CGH analysis using CGH Cytoscan 750 K array detected a 7q34 deletion (*PRSS3P2* gene), (**E**) Array-CGH analysis also detected the 17q21.31 gain (*KANSL1* gene). (**F**) IHC showed positive p53 nuclear expression of bone marrow cells (×200). (**G**) Hematoxylin and eosin (H&E) stains atypical megakaryocytes, micromegakaryocytes (white arrows), and megaloblastic changes in erythroid cells (black arrows) (×400). (**H**) CD61 immunostaining, highlights a small cluster of micromegakaryocyte outlined by the antibody (×200).

A 4-year-old girl was admitted to Instituto de Pediatria e Puericultura Martagão Gesteira, Universidade Federal do Rio de Janeiro, Brazil, in March 2010, due to recurrent infections and severe neutropenia. The main clinical events and laboratory tests performed were summarized in a timeline of 12-year follow-up (Supplemental Figure S1). At admission, peripheral blood (PB) analysis showed: hemoglobin 9.8 g/L, platelet count 234 × 10^9^/L, and white blood cell count (WBC) 6.9 × 10^9^/L with neutrophils count: 0.56 × 10^9^/L. The bone marrow (BM) presented: hypocellularity with a granulocytic maturation arrest and micromegakaryocytes without fibrosis. BM immunophenotyping using the EuroFlow panel and settings [14] showed granulocytic maturation blocked with the absence of CD10 expression in mature neutrophils, together with CD13 and CD64 overexpression in promyelocytes and in the whole granulocytic maturation, respectively. Further, both monocytes and neutrophils abnormally expressed CD56 (Supplemental Figure S2). The cytogenetic analysis by G-banding and fluorescence in situ hybridization showed: 46,XX,t(3;8)(p26;q21)c [25] (Figure 1A). The patient was classified as cMDS with low blasts (cMDS-LB) [1]. Prophylactic antibiotic therapy with amoxicillin was initiated in May 2010. In April 2013, the patient had <100 neutrophils and started granulocyte colony-stimulating factor (GCSF), which sustains neutrophil levels. In 2014, the patient had clinical worsening with marked hypoplasia of all hematopoietic lineages, a decrease in G:E ratio (2:1) with megaloblastic changes in erythroid cells lineage, and the presence of micromegakaryocytes. Immunophenotyping showed: granulocytic and erythroid maturation blocked and excess of blasts (5.4%) featuring a cMDS with an increased blast (cMDS-IB). Progressively, clinical evolution had been getting worse with very severe neutropenia (<0.2 × 10^9^/L) without response to GCSF. In 2018, the patient had granulocytic maturation with intense blockage, more erythroid cells, dysplastic megakaryocytes, and 8% of myeloid blasts, characterizing the progression of the disease. At this time, allogeneic hematopoietic stem cell transplantation (aHSCT) was indicated. Then, search in the family identified the father as a donor for aHSCT. Nevertheless, he also has the t(3;8)(p26;q21)c change. A search for an international donor found an unrelated donor, but he was not available. During this period, when we were at the peak of the COVID-19 pandemic, the patient began to show clinical improvement and the stabilization of the number of blast cells. Since 2020, the patient has had no clinical complications and continues to use G-CSF. The patient had the stabilization of the blast cells count through the sequential bone marrow’s analysis. Since 2020, the patient has had no clinical complications. In 2022, the hemogram showed: hemoglobin 12.4 g/L, platelet count 242 × 10^9^ L, and WBC 4.47 × 10^9^ L. The patient remained under observation and using G-CSF and clinical follow-up semi-annual. No changes in the BM analysis were in the scheme of “watching and waiting” for a possible HSCT.

## 3. Methodology and Results

### 3.1. Conventional and Molecular Cytogenetics

Cytogenetic analyses were performed in BM cells from cultures in RPMI 1640, with 20% fetal calf serum (GIBCO) at 37 °C for 24 h [15]. For the constitutional karyotype, the PB cells were cultured with the same medium and serum, with the addition of 0.02 µg/mL of phytohemagglutinin (PHA) at 37 °C for 72 h. Cell cultures from BM and PB were pulsed with colcemid to a final concentration of 0.05 µg/mL for the final hour of incubation. Cells were harvested by standard procedures, with the hypotonic shock using 0.075 M KCl for 20 min, and finally, the cells were fixed in methanol:acetic acid (3:1). The chromosomal analyses were performed using G-banding. Chromosomes were identified and arranged according to the International System from Human Cytogenomic Nomenclature (ISCN) 2020 criteria [16]. The constitutional chromosomal alteration (CCA) was characterized by a whole chromosome painting (WCP) probe for chromosomes 3 and 8, and also the partial chromosome painting (PCP) probe for the 3q arm showed that it was a non-reciprocal translocation (Vysis, Abbott Laboratories, Illinois, USA) (Figure 1B,C). The hereditary origin of t(3;8)(p26;q21) was confirmed in 100% of her father’s, grandmother’s, and uncle’s karyotypes. Couples in which one of the partners had the translocation self-reported difficulty in becoming pregnant, self-reported the occurrence of spontaneous abortions, and denied a family history of hematological disease. Clinical genetic exams showed a normal phenotype in the patient with MDS.

In order to investigate possible cytogenomic alterations associated with the development of MDS, we analyzed the presence of cryptic chromosomal alterations using the CytoScan 750 K Array (Affymetrix, California, USA) in the patient sample. The DNA was extracted using the QIAmp DNA Mini Kit (Qiagen, Hilden, Germany). The array-CGH data were scanned and analyzed using CytoScan 750 K Array according to the instructions of the manufacturer. A small deletion at chromosome 7q34 (~10 kb), involving the *PRSS3P2* gene (Figure 1D), as well as a gain at chromosome 17q21.31 (~40 kb) in the *KANSL1* gene, was found in the patient (Figure 1E).

Cytogenetic molecular abnormalities associated with Familial MDS-AML were also searched using the Multiplex Ligation-dependent Probe Amplification (MLPA) for Familial MDS/AML, MLPA SALSA P437_B1 probe sets (MRC Holland, Amsterdam) in the proband, father, and mother samples, according to the manufacturer’s instructions. Sequences were analyzed by Coffalyser in combination with the SALSA MLPA kit-specific Coffalyser paste (MRC Holland, Amsterdam). Nevertheless, neither of them had alterations related to familial MDS/AML using this test.

### 3.2. Immunohistochemistry

The patient’s immunohistochemistry (IHC) stain showed a low level of CD34+ cells (1%) and negativity to p53 expression in BM biopsy at diagnosis. During the patient’s clinical progression to cMDS with an increased blast (cMDS-IB), a strong nuclear positivity for p53 (4–5% of cells) was observed (Figure 1F). Otherwise, BM had atypical megakaryocytes with micromegakaryocytes and megaloblastic and dyserythropoietic changes in nucleated red blood cells (Figure 1G,H).

### 3.3. Next-Generation Sequencing

**Figure 2:**
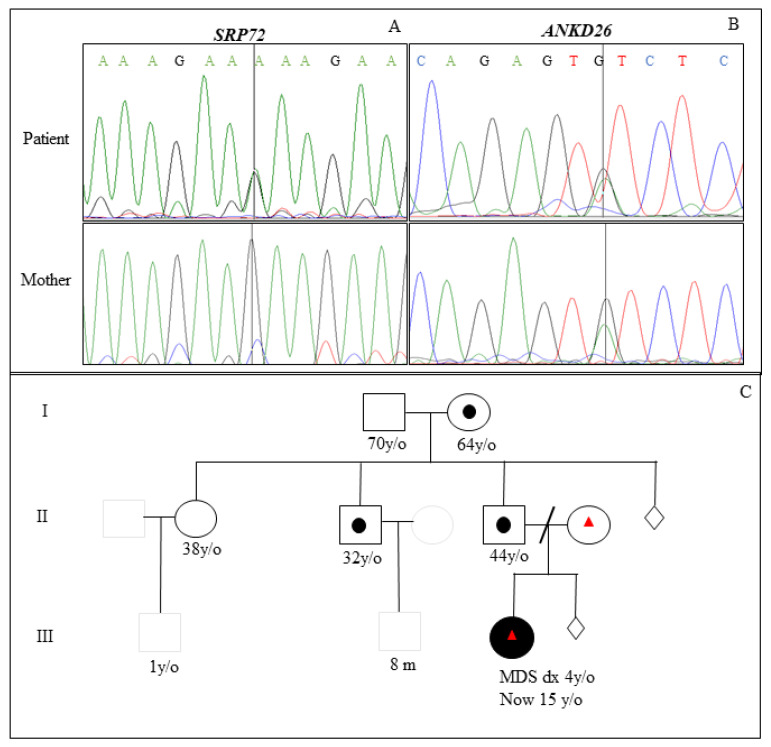
Sanger sequencing of the *SRP72* and *ANKRD26* genes and three-generation pedigree. (**A**) The sequencing of *SRP72* gene on the genomic DNA of PB observed the origin of the maternal (homozygous) germline variant in the patient (heterozygous). (**B**) Sequencing of *ANKRD26* gene on the genomic DNA of PB showed the maternal germline variant in the patient (heterozygous). (**C**) Three-generation pedigree of cMDS patient showing that t(3;8)(p26;q21)c was detected in patient, father, uncle, and grandmother. MDS patient also had maternal germline variants in *SRP72* and *ANKRD26* genes. Squares and circles are symbols commonly used to construct a pedigree following human genetic nomenclature.

In search of germline alterations with a possible familial association, a custom panel for next-generation sequencing (NGS) was done for the Ion Torrent Personal Genome Machine (PGM) platform (Life Technologies) for genes: *GATA2*, *RUNX1*, *CEBPA*, *ANKRD26*, *ETV72*, *SAMD9*, *SAMD9L*, *PTPN11*, *NRAS*, *SETBP1*, *DDX41*, *TP53*, *FLT3*, *SRP72*, and *JAK3* covering all regions of reduction, achieving >90% coverage. Genomic DNA (20 ng) was used for Library preparation using the Ion AmpliSeq Library Kit 2.0. Adapters containing barcodes were added to each sample (Ion Xpress Barcode Adapters, Thermo Fisher Scientific, MA, USA). Libraries were purified and quantified by real-time PCR using the Ion Library TaqMan Quantification kit (Thermo Fisher Scientific, MA, USA). Emulsion PCR was performed on the Ion One Touch 2 Instrument using the Ion PGM Hi-Q-View OT2 Kit. The enrichment of the library was performed with Ion Sphere Particles (ISPs) in the Ion One Touch ES equipment (Thermo Fisher Scientific). Libraries were loaded onto the Ion 316 v2 chip and sequenced on the Ion Torrent PGM sequencer (Thermo Fisher Scientific). All procedures were made according to the manufacturer’s recommendation. Ion Reporter software v.5.20 (Available online: https://ionreporter.thermofisher.com (accessed on 2 April 2023)) was used for alignment and sequence analysis. The index patient’s PB showed two germline variants in the *ANKRD26* and *SRP72* genes (Table 1). These genetic variants were confirmed by Sanger sequencing, and they were also identified in the mother (Figure 2A,B). We constructed the familial pedigree with the collections of the cytogenetics and genomics results (Figure 2C).

## 4. Discussion and Conclusions

CCA may confer genetic instability and not be directly associated with neoplasia development, being necessary for other alterations. Nevertheless, CCA may increase the risk of miscarriage or transmitting chromosomal abnormalities to offspring with developmental disabilities [3,17]. Interestingly, in our study, genetic instability due to t(3;8)(p26;q21)c was also associated with a family history of spontaneous abortions. However, only one family member developed MDS, suggesting the necessity of the acquisition of other cytogenetics and molecular abnormalities during cMDS development.

Cryptical chromosomal alterations involving *PRSS3P2* and *KANSL1* genes were observed by array-CGH contributed to the development of MDS. *PRSS3P2* is a trypsinogen gene located at the T cell receptor beta locus that is a mutational hotspot [18]. Interestingly, a long non-coding RNA (lncRNAs) can also be encoded in this region of gene *PRSS3P2*. This lncRNA has multiple regulatory functions at all levels of gene expression [19], which may be contributing to the MDS clinical evolution. Dysregulation of lncRNAs has previ-ously been described in patients with MDS [20].

The KANSL1 gene is a subunit of the nonspecific lethal complex (NSL) 7 and the mixed lineage/domain-defined leukemia (MLL/SET) complex. The *KANSL1* gene has been recognized as a cancer driver involving epigenetic regulation by acting in the unpacking of the chromatin structure, altered transcription, and controlling the H3 (Lys4) methylation of the cis-regulatory gene activation regions [21,22]. Acting in the acetylation process is necessary for the transcriptional activation of *TP53* [23]. The overexpression of p53 is associated with a more aggressive clinical outcome and *TP53* gene mutation [24]. In our study, no pathogenic genetic variants were observed in the *TP53* gene using the NGS analysis. Thus, our results suggest that the p53 overexpression during disease evolution was a result of epigenetic dysregulation caused by the *KANSL* gene.

Our patient, in addition to t(3;8)(p26;q21)c, also presented genetic variants in the *ANKRD26* and *SRP72* genes of maternal origin. *ANKRD26* gene acts on the regulation of three myeloid lineages modulating the activity of three type I cytokine receptors that are essential in normal hematopoiesis [25]. Our patient presented dysplasias in the erythroid, granulocytic, and megakaryocytic lineages during the MDS progression. *ANKRD26* gene variants are related to thrombocytopenia 2 and predisposition to myeloid neoplasms [26]. The patient had a missense variant and did not develop thrombocytopenia, whereas the literature has shown that the 5’UTR is a known hotspot in the *ANKRD26* and has low penetration for the development of myeloid neoplasms but has an 8% of lifetime risk. [4,6]. The *ANKRD26* gene also has low penetration for the development of myeloid neoplasms, [4]. *SRP72* variants contribute to aplastic anemia and MDS, being described as a new category of genes altered in neoplasms by acting in the translocation and processing of proteins [27]. Until now, little has been known regarding the incidence of *SRP72* variants and the risk for hematologic malignancies due to the rarity of these germline variants [7]. The patient had a synonymous variant considered benign, so it probably did not solely contribute to the development of cMDS. In the mice model, the heterozygous loss of *SRP72* is not associated with major hematological phenotypes [7]. It is interesting to note that the patient had an unusual timeline with 13 years of following up cMDS. The penetrance of the germline gene variants is heterogeneous [3,4]. The *ANKRD26* rs12359281 variant has been seldomly described. We believe that the multi-hits observed in the patient are random and not enough for MDS transformation. The search for the String-db platform did not identify any interaction between *ANKRD26* and SRP72. The *ANKRD26* variant might affect the expression of granulocyte differentiation 24. In contrast, the SRP72 rs34419325 might act as modulating the apoptosis [7,27]. Therefore, we suppose that the effect of both variants can interfere in the subsets blood cells’ production. Both *PRSS3P2* and *KANSL1* act on epigenetic regulation, and these mechanisms can overlap and be reversible by antagonistic agents. This could partly explain the condition of the patient who remained stable. The effect of *KANSL* on p53 is probably not enough to explain the whole picture. Probably, the patient’s clinical picture is the result of all these alterations together.

The aHSCT is considered the only potentially curative treatment option for MDS patients. It is recommended for patients with severe clinical complications and poor prognoses. The relapse is a major cause of failure post-HSCT and is generally associated with poor outcomes [28,29]. In this patient, aHSCT was indicated. However, the search for donors in the family identified the father as a donor. He also has the t(3;8)(p26;q21)c variant. The unrelated donor found in an international search declined to donate. During this period, we were at the peak of the COVID-19 pandemic. Fortunately, the patient began to show clinical improvement with the stabilization of the number of blast cells.

In conclusion, the present study reports for the first time the history of multiple hits associated with the development and clinical evolution of cMDS: a t(3;8)(p26;q21)c of paternal origin, *ANKRD26* and *SRP72* variants of maternal origin, and also the acquisition of alterations in *PRSS3P2* and *KANSL* genes. *KANSL* gene possibly activated the abnormal expression of p53 contributing to the development and cMDS progression. The use of cytogenetic and genomic tests was fundamental for the familial history and the identification of the genetic predisposition to cMDS. This study shows the importance of cMDS in investigating the family nucleus for genetic counseling, also to avoid a potential donor from the family with the same genetic abnormalities for the hematopoietic stem cell transplantation and for a better understanding of the pathogenesis of childhood MDS.

## Figures and Tables

**Table 1:** Description of germline variants present in cMDS.

Molecular Data *	Variants of Genes	
	*SRP72*	*ANKRD26*
Reference Genome	ENSG00000174780	ENST00000376087.4
Locus	chr4:57361553	chr10:27353007
dbsnp	rs34419325	rs12359281
Impact	Benign	Moderate
Consequence	Synonymous variant	missense_variant
Genotype	A/G	ATC/GTC
Allele_coverage	A = 526, G = 539	T = 992, C = 1008
Allele_ratio	A = 0.4939, G = 0.5061	T = 0.496, C = 0.504
Allele_frequency (%)	50.61	50.40
MAF	0.07645	0.067
Transcript	NM_006947.4	NM_014915.3
Protein	p.Lys557=	p.Ile425Val
Coding	c.1671A>G	c.1273A>G
Related disease	Aplasia and myelodysplasia	Thrombocytopenia 2

dbsnp: Single Nucleotide Polymorphism Database; MAF: Minor allele frequency; chr: chromosome. * For the mutations molecular description were used the Ion Reporter software (Available online: https://ionreporter.thermofisher.com/ (accessed on 2 April 2023)), Ensembl Variant Effect Predictor (VEP) (Available online: https://www.ensembl.org/Tools/VEP (accessed on 2 April 2023)), Varsome (2022) (Available online: https://varsome.com/ (accessed on 2 April 2023)).

## Data Availability

All data analyzed during the current study are available from the corresponding author (T.d.S.F.) on reasonable request.

## Supplementary Materials

The following supporting information can be downloaded at: https://www.mdpi.com/article/10.3390/jcm12093171/s1, Figure S1: The main clinical events and laboratory tests are summarized in a timeline of a 12-year follow-up. Figure S2: Sequential BM immunophenotypic analysis by flow cytometry.
