# Supplementary material for: A Novel Constitutional t(3;8)(p26;q21) and ANKRD26 and SRP72 Variants in a Child with Myelodysplastic Neoplasm: Clinical Implications
Source: J Clin Med. 2023 Apr 28;12(9):3171. doi: 10.3390/jcm12093171 (PMC10179081; doi:10.3390/jcm12093171)
Supplement: Supplementary file 1 [file jcm-12-03171-s001.zip › jcm-2325110-supplementary.pdf]

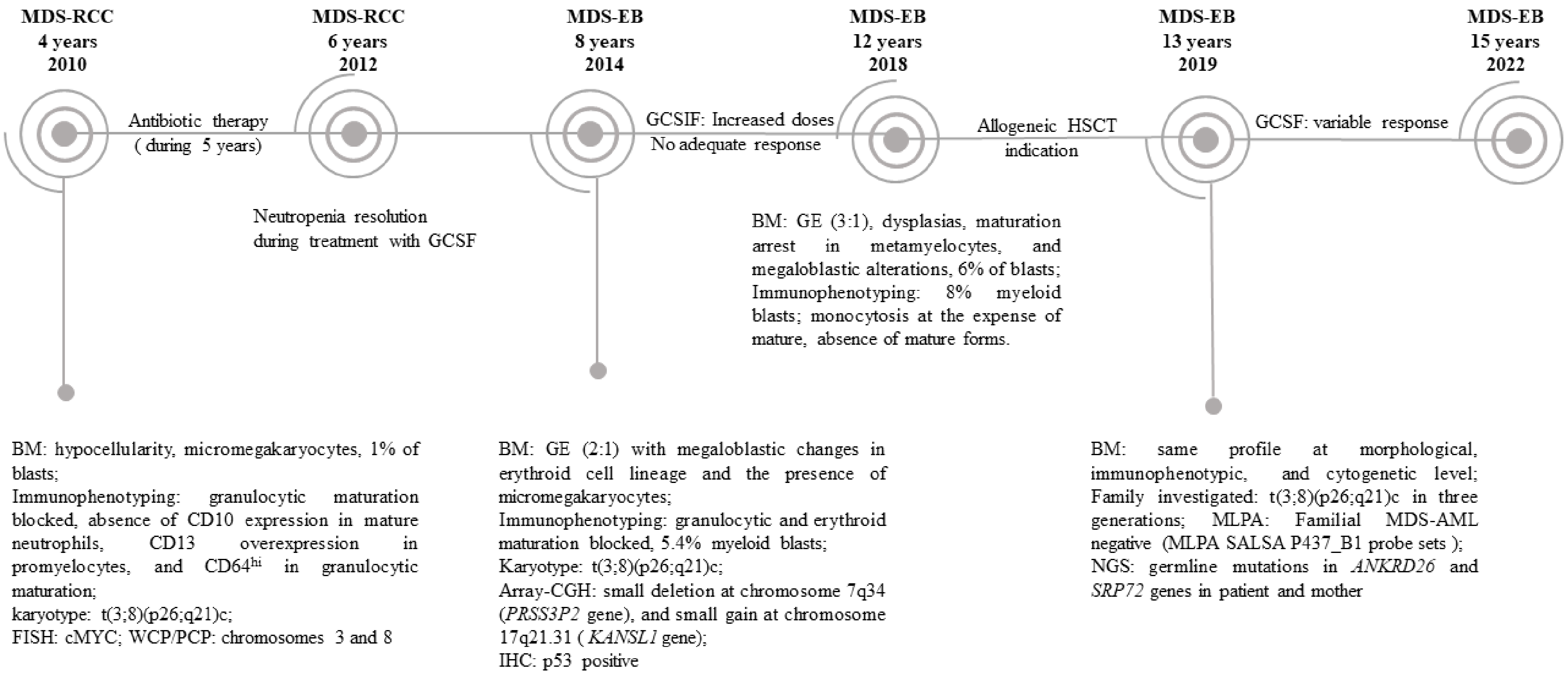

Supplemental Figure S1: The main clinical events and laboratory tests are summarized in a timeline of a 12-year follow-up.

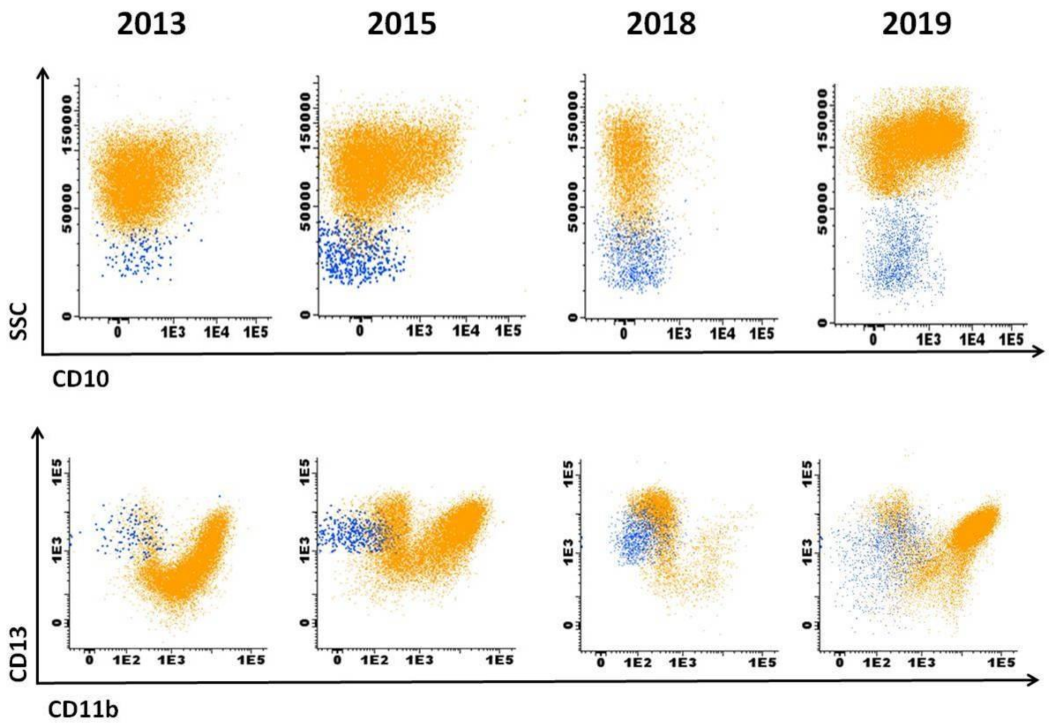

Supplemental Figure S2: Sequential BM immunophenotypic analysis by flow cytometry.

A blocked granulocytic maturation (orange dots) with a progressive increase of neutrophil immature precursors (blue dots). In 2013, we observed a predominance of intermediate subpopulations of granulocytic lineage, as myelocytes/metamyelocytes, in absence of mature CD10+ neutrophils. In 2015, the blocked granulocytic maturation evolved with a decrease of such intermediate neutrophils subpopulations, but more mature neutrophils were CD10-/+lo. In 2018, an increase of myeloblasts and a predominance of more immature neutrophil precursors began and intensified in 2019, with an excess of myeloid blasts. Conversely, more mature neutrophils recovered the expression of CD10.
